# Waffle-Like Carbons Combined with Enriched Mesopores and Highly Heteroatom-Doped Derived from Sandwiched MOF/LDH/MOF for High-Rate Supercapacitor

**DOI:** 10.3390/nano10122388

**Published:** 2020-11-30

**Authors:** Szu-Chen Wu, Po-Hsueh Chang, Syun-Hong Chou, Chih-Yang Huang, Ta-Chung Liu, Cheng-Hsiung Peng

**Affiliations:** 1 Department of Materials Science and Engineering, National Chiao Tung University, 1001 University Road, Hsinchu 300, Taiwan; s320431@hotmail.com (S.-C.W.); a0143499@gmail.com (S.-H.C.); bonbon19940827@gmail.com (C.-Y.H.); 2 Department of Chemical and Materials Engineering, MingHsin University of Science and Technology, 1 Xinxing Road, Xinfeng, Hsinchu 304, Taiwan; pohsueh.chang@gmail.com

**Keywords:** supercapacitors, waffle-like nanoporous carbon, nitrogen-doped, sieving effect, metal-organic framework

## Abstract

Supercapacitors (SCs) are promising for powering mobile devices, electric vehicles and smart power grids due to their fast charge/discharge rate, high power capability and robust cycle stability. Nitrogen-doped porous carbons are great alternatives because they provide pseudocapacitance without losing their power rate. Nanoporous carbon derived from metal organic frameworks (MOFs) is an ideal precursor for preparing heteroatom-doped carbons due to their abundant nitrogen contents and incredible specific surface areas. However, severe aggregations and the leakage of nitrogen can occur during harsh carbonization. In this study, we used CoAl-LDH (cobalt aluminum layered double hydroxide) as an in-situ growth substrate, allowing Co-based MOF to uniformly grow onto the CoAl-LDH to form a sandwiched MOF/LDH/MOF structure. After acid etching, we obtained waffle-like nanoporous carbons (WNPC). WNPC exhibited high nitrogen and oxygen retention (7.5 wt% and 9.1 wt%) and a broad mesopores distribution with specific surface areas of 594 m^2^g^−1^, which promoted a sieving effect. This renders a specific capacitance of 300.7 F·g^−1^ at 1 A·g^−1^ and the high retention (72%) of capacitance at 20 A·g^−1^, ensuring its use at high-rate supercapacitor electrodes. Finally, the WNPC symmetric supercapacitor reaches a superior specific energy of 27 W·h·kg^−1^ at a power of 500 W·kg^−1^, and a good cycle stability (85% capacitance retention after 10,000 cycles).

## 1. Introduction

Supercapacitors (SCs) are promising for powering mobile devices, electric vehicles and smart power grids due to their fast charge/discharge rate, high power capability, excellent reversibility, and robust cycle stability [1,2,3,4]. Nevertheless, the poor energy density of SCs limits their practical application compared to most lithium-ion based batteries [5,6,7]. Current strategies have primarily focused on hybridization of carbonaceous materials with pseudocapacitive materials for massive charge production by redox reactions, as well as increasing specific surface areas of carbons. Although many pseudocapacitive materials such as nano metal oxides (e.g., NiMoO_4_ and NiCo_2_O_4_) [8,9,10,11,12] and conductive polymers (e.g., polyaniline and polypyrrole) [13,14] have been proposed, these pseudocapacitive materials suffer from low electrical conductivity, high costs, and poor cycle stability [8,9,10,11,12,13,14]. Recently, nitrogen-doped (N-doped) carbon materials have attracted considerable attention because they not only maintain the electrochemical performance of electric double-layer capacitors (EDLCs) with excellent cycle stability, but also provide pseudocapacitance without compromising power rate [15,16].

As a class of metal organic frameworks (MOFs), synthesized through the coordination reaction of transition metal ion (such as Co^2+^, Zn^2+^ and Cu^2+^ etc.) and imidazole organic compounds, N-doped carbon materials are considered an ideal precursor for preparing pseudo-EDLCs due to their abundant nitrogen contents (over 34 wt% of nitrogen in imidazole organic ligands). Furthermore, MOFs exhibit an extreme high specific surface area exceeding 1300 m^2^g^−1^ due to their intrinsic microporosity. Yet their use in EDLCs has been limited for nanoporous carbons via pyrolysis, leading to a much lower electrical conductivity than that of graphitic carbons. Although higher electrical conductivity can be enhanced by graphitization at a higher temperature pyrolysis for carbonized MOFs, N-doped yields are less preserved due to low C-N binding energy [17], and carbonized MOFs are usually accompanied with severe aggregation, which vastly decreases the specific surface areas and hampers the diffusion of electrolytes.

Recently, different metal ions have been adopted as MOF coordinators to adjust the electrical conductivity and N-doped content. For example, Salunkhe et al. developed a porous carbon by direct pyrolysis of Zn-MOF (Zn-based MOF) to prepare high N-doped porous carbons with a high specific surface area of 1523 m^2^g^−1^ [18]. Torad et al. utilized Co-MOF (Co-based MOF) as a precursor for preparing supercapacitor electrodes to produce a well-conducting porous carbon, which resulted in high graphitization of carbons because of the strong catalytic ability of metallic Co atoms [19]. However, the N-doped content for Co-MOF is only about 5.7 wt%, much lower than that of carbonized Zn-MOF (16.1 wt%). The N-doped content and the electrical conductivity is generally contradictory.

Other studies have reported that introducing a template for MOFs growth is a promising way to produce dispersive MOFs and to maintain the structural integrity of MOFs, thus preventing collapse and aggregation under harsh pyrolysis. Yin et al. proposed a hybridized nanoporous carbon combined with Co-MOF on graphene oxide (GO) sheets via electrostatic self-assembly [20]. This composite showed a better electrochemical performance due to the well-ordered porous structure. Pachfule et al. used polyfurfuryl alcohol (PFA) as a soft template for MOFs growth to effectively improve agglomeration after carbonization [21]. Despite the use of the above strategies to address aggregation issues, there is still a trade-off between the electrical conductivity and the N-doped yields. Therefore, it is very challenging to simultaneously optimize electrical conductivity, N-doped yield, and a well-ordered porous nanostructure for carbonized MOFs.

In this study, we utilized dual-atom cobalt aluminum layered double hydroxide (CoAl-LDH) as a synthetic template for the in-situ growth of Co-MOF onto CoAl-LDH to form a sandwiched MOF/LDH/MOF hybrid. Because the well-ordered Co atoms on CoAl-LDH can serve as nucleation and growth active sites for the in-situ growth of Co-MOF, the strong covalent chemical bonding between CoAl-LDH and Co-MOF maintains the structural integrity and homogeneity under harsh pyrolysis at 800 °C to give an ordered porous nanostructure. Moreover, the strong catalytic ability of metallic Co atoms in CoAl-LDH and Co-MOF leads to a high graphitization [17], which is believed to provide MOF/LDH/MOF with an enhanced electrical conductivity. This hybridized MOF/LDH/MOF possesses a high level of N (7.5 wt%) and O (9.1 wt%), higher than that of the nanoporous carbon (NPC) derived from carbonized Co-MOF (3.4 wt% of N and 2.5 wt% of O), and exhibits a specific surface area of 594 m^2^g^−1^ which is larger than NPC of 394 m^2^g^−1^. With the help of the broad distribution of mesoporosities, waffle-like nanoporous carbons (WNPC) showed better combined micro/mesoporous characteristics to promote good kinetics for electrolyte migration. The synergistic effect of using dual-atom CoAl-LDH as an in-situ growth template combined with the favorable survival of heteroatom-doping and a micro/mesoporous characteristics, produced excellent electrochemical performance with a specific capacitance of 300.7 F·g^−1^ at 1 A·g^−1^ for WNPC. Finally, the as-fabricated WNPC symmetric supercapacitor exhibits a superior specific energy of 27 W·h·kg^−1^ at a specific power of 500 W·kg^−1^, and a decent cycle stability (85% capacitance retention after 10,000 cycles).

## 2. Experimental Section

### 2.1. Chemicals and Reagents

Cobalt (II) nitrate hexahydrate (Co(NO_3_)_2_·6H_2_O; 99%) and Aluminium (III) nitrate enneahydrate (Al(NO_3_)_3_·9H_2_O; 99%) were purchased from Showa Co. Ltd., Tokyo, Japan). Urea (99%) was purchased from J.T.Baker Co. Ltd. (Delaware, PA, USA). 2-methylimidazole (2-melm; 99%) and phosphoric acid (H_3_PO_4_; 85%) were purchased from Acros Co. Ltd. (Morris Plains, NJ, USA). Methanol was purchased from ECHO CHEMICAL Co., LTD (Miaoli, Taiwan). Hydrochloric acid (HCl; 37%), polyvinylidene difluoride (PVDF; MW: 200,000), N-methyl 2-pyrrolidine (NMP), propylene carbonate (PC; 99.7%), and carboxymethyl cellulose (CMC) were purchased from Sigma Aldrich Co. Ltd. (St. Louis, MO, USA). Carbon black was purchased from Cabot Co., Ltd. (Boston, MA, USA). Sulfuric acid (H_2_SO_4_, 95–98%) was purchased from Scharlau (Barcelona, Spain). Styrene-butadiene rubber (SBR) was purchased from Scientific Polymer Products, Inc (Ontario, NY, USA). Tetraethylammonium tetrafluoroborate (TEABF_4_; 99%) was puberchased from Alfa Aesar Co. Ltd. (Miaoli, Taiwan).

### 2.2. Preparation of CoAl-LDH

Typically, we dissolved Co(NO_3_)_2_·6H_2_O (10 mmoL), Al(NO_3_)_3_·9H_2_O (5 mmoL), and urea (100 mmoL) in 50 mL of distilled water (DI water), and then transferred the mixture into a Teflon-lined stainless steel autoclave. Next, the autoclave was sealed and heated at 110 °C for 24 h. After cooling the reaction system, the resulting product was centrifuged and washed with deionized water for several times and finally freeze dried.

### 2.3. Synthesis of Co-MOF

In synthesizing Co-MOF, we prepared two solutions (solution A and solution B). Solution A was prepared by dissolving 1.45 g (0.1 M) of Co(NO_3_)_2_·6H_2_O in 50 mL of methanol and solution B was prepared by dissolving 3.28 g (0.8 M) of 2 mL in 50 mL of methanol. Subsequently, solution B was quickly added to solution A and stirred for 15 min. After keeping undisturbed for 15 min at room temperature, the reaction mixture was centrifuged several times with methanol to obtain Co-MOF nanoparticles. Co-MOF nanoparticles were dried overnight at 80 °C oven for 24 h.

### 2.4. Synthesis of Sandwiched MOF/LDH/MOF

Typically, 50 mL of Co(NO_3_)_2_·6H_2_O methanol solution (0.1 M) as a reagent was added into a 50 mL methanol solution containing 0.1 g as-prepared freeze-dried CoAl-LDH, and then a 2-methylimidazole (2-melm) methanol solution (50 mL, 0.8 M) as an initiator was rapidly poured into the above solution under magnetic stirring at room temperature for 15 min to obtain the MOF/LDH/MOF resulting solution. The resulting solution was centrifuged several times with methanol to obtain MOF/LDH/MOF powders and dried overnight at 80 °C oven for 24 h.

### 2.5. Preparation of Waffle-Like Nanoporous Carbon (WNPC) and Nanoporous Carbon (NPC)

The as-prepared Co-MOF and MOF/LDH/MOF were directly carbonized with a heating rate of 5 °C·min^−1^ under flowing nitrogen gas at 800 °C for 3 h and then cooled down to room temperature. The carbonized samples of MOF/LDH/MOF and Co-MOF were defined as C_M/L/M_ and C_Co-MOF_, respectively. C_M/L/M_ and C_Co-MOF_ were each collected and carefully washed with a mixture acid solution containing HCl, H_3_PO_4_ and DI water aqueous solution (1:3:6) to remove the metal oxides at 90 °C for stirring 24 h, followed by washing 4 times with distilled water and then dried under vacuum conditions for 24 h at 60 °C. The C_M/L/M_ and C_Co-MOF_ after acid etching were defined as WNPC and NPC, respectively.

### 2.6. Materials Characterization

X-ray diffraction (XRD) data were collected with a D2 theta-theta diffractometer (PerkinElmer Co. Ltd., Taipei, Taiwan) using Cu-Kα radiation in a standard two-circle X-ray diffractometer operated at 30 kV and 10 mA and recorded for two theta values between 5° and 40°. MOF/LDH/MOF powders were pressed into a plate and then observed using a section dual-beam focus ion beam microscope (FIB, LYRA3, TESCAN, Brno, Czech Republic). As-prepared particle morphology was examined by field emission scanning electron microscopy and energy dispersive X-ray spectrometer (FESEM&EDX, JEOL-6700, JEOL, Tokyo, Japan) and field emission gun transmission electron microscopy and energy dispersive X-ray spectrometer (FEG-TEM&EDX, JEM-2100 and JEM-F200, (JEOL, Tokyo, Japan). X-ray photoelectron spectroscopy (XPS) data were collected using a Microlab 350 system (VG Scientific, Hsinchu, Taiwan) with monochromatized Al-Kα X-ray source. The Raman spectra was obtained using a HR800 system (HORIBA Jobin Yvon, Kyoto, Japan) equipped with an Ar laser (λ = 514.5 nm). Nitrogen adsorption–desorption isotherms were collected on an ASAP 2020 (Micromeritics Instrument Co. Norcross, GA, USA) at 77 K. Pore size distributions were calculated from the N_2_ adsorption data using the Brunauer, Emmett and Teller (BJH) for 2 nm above of pores, and Horvath–Kawazoe (H–K) method (slit-pore) for 2 nm below of pores. Zeta potentials were measured in water with pH values of 7 using a Zetasizer (Delsa Nano C, Beckman Coulter Inc., Brea, CA, USA).

### 2.7. Electrochemical Measurements

The electrochemical measurements were conducted with a three-electrode system using Pt as the counter electrode and Ag/AgCl as the reference electrode in 1 M H_2_SO_4_ solution. The working electrode was prepared by mixing 90 wt% of as-prepared porous carbons, 5 wt% of carbon black and 5 wt% of polytetrafluoroethylene in 1 mL NMP to form a slurry. The resulting slurry was homogenously ultra-sonicated and coated onto a glassy carbon round disk electrode (5 mm in diameter) with 5 μL containing 25 μg of electroactive materials, followed by drying at 90 °C. Cyclic voltammograms (CV) were performed using an electrochemical workstation (CHI 6278E CH Instruments, USA) in the voltage range of 0 to 1 V. For the galvanostatic charge–discharge (GCD) measurements, the charge–discharge current density based on the mass loading of the electrodes was varied from 4 A·g^−1^ to 20 A·g^−1^. to evaluate the specific energy (SE) and specific power (SP) of the as-prepared porous carbon electrode. The specific capacitance C as F·g^−1^, SE as W·h·kg^−1^, and SP as W·kg^−1^ were calculated from the chronopotentiometric curves using the following Equation (1).

(1) $$C=\frac{I\times ∆t}{m\times ∆V}$$

Here, *I* is the charge–discharge current at a discharge time Δ*t* (s), Δ*V* is the potential window, and *m* is the mass of active material on the working electrode.

For full cell electrochemical tests, the electrodes were prepared by coating a slurry containing the 91 wt% of WNPC, 2.5 wt% of carbon black, 1.5 wt% of CMC, and 0.5 wt% of SBR on Al foil. The electrochemical performance of the symmetric supercapacitors was examined by assembling CR2032 coin-type cells with cellulose (NKK TF4840) in a dry room. The loading level for one single electrode layer was 0.0942 mg cm^−3^ (the thickness of one single electrode layer was ∼65 µm and the area of one single electrode layer was ~1.3 cm^2^). The electrolyte used was 1 M TEABF_4_/PC. In two-electrode measurements, the gravimetric capacitance values of the symmetric device were calculated using the GCD method according to the following Equation (2):

(2) $$C_{\mathrm{cell}}=\frac{I\times ∆t}{2\times m\times ∆V}$$

Here, *I* is the charge–discharge current at a discharge time t (s), Δ*V* is the potential window, and *m* is the mass of the active material of one single electrode layer.

The specific energy (SE) and specific power (SP) were calculated from Equations (3) and (4), respectively.

(3) $$\mathrm{SE}=\frac{\frac{1}{2}C_{\mathrm{cell}}V^{2}}{3.6}$$

(4) $$\mathrm{SP}=\frac{3600\times SE}{t}$$

## 3. Results

### 3.1. In-Situ Growth of Co-MOFs on CoAl-LDH Sheets

**Scheme 1:**
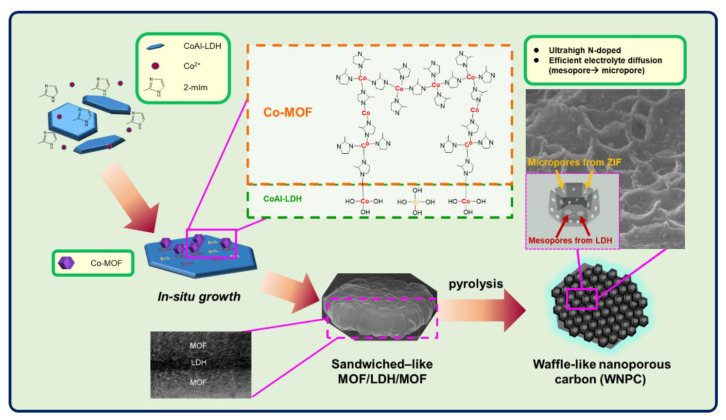
Schematic view of the synthesis of the sandwiched MOF/LDH/MOF via in-situ growth and the waffle-like nanoporous carbon (WNPC).

In this study, we utilized dual-atom cobalt aluminum layered double hydroxide (CoAl-LDH) as a synthetic template for in situ growth of Co-MOF onto CoAl-LDH to form a sandwiched MOF/LDH/MOF structure hybrid. Then, the MOF/LDH/MOF under harsh pyrolysis at 800 °C to give ordered porous nanostructure. After metallic template removal, abundant and dispersive mesoporosites remained and a waffle-like nanoporous carbon (WNPC) was obtained (Scheme 1).

**Figure 1:**
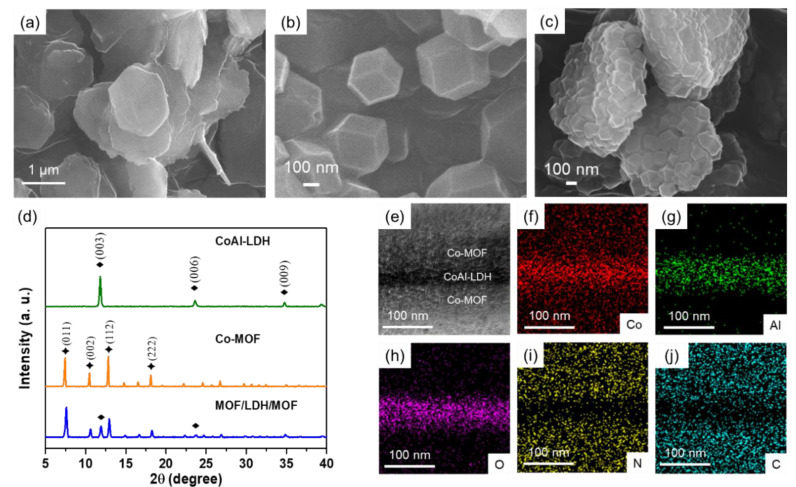
SEM images of (**a**) cobalt aluminum layered double hydroxide (CoAl-LDH); (**b**) Co-based metal organic frameworks (Co-MOF) and (**c**) MOF/LDH/MOF; (**d**) XRD patterns of CoAl-LDH, Co-MOF and MOF/LDH/MOF; (**e**) The cross-sectional TEM images of MOF/LDH/MOF and (**f**–**j**) the corresponding element mapping images of Co, Al, O, N, and C.

Figure 1a,b shows the SEM images of CoAl-LDH and Co-MOF, respectively, as comparisons with the as-prepared sandwiched MOF/LDH/MOF hybrid. In our work, CoAl-LDH sheets displayed typical hexagonal shapes with average size of about 1.7 ± 0.2 µm and a thickness of 84.7 ± 10.3 nm (Figure 1a), additionally, Co-MOFs also show typical rhombic dodecahedral crystals with an average particle size of about 341.9 ± 11.7 nm (Figure 1b). For the MOF/LDH/MOF hybrid, owing to the well-ordered Co atoms on CoAl-LDH serving as nucleation and growth sites, an in-situ coordination reaction occurs on the surface of CoAl-LDH and then generates a sandwiched MOF/LDH/MOF hybrid where Co-MOF crystals are strongly chemically-bonded and well dispersed on the CoAl-LDH (Figure 1c and Figure S1). In order to examine the homogeneity of the MOF/LDH/MOF hybrid, we compared the MOF/LDH/MOF (via in-situ growth) with the mixture of CoAl-LDH and Co-MOF powders (via direct mixing). After centrifugation, the MOF/LDH/MOF presented homogeneous powders without layer separation (Figure S2a), while the mixture of Co-MOF and CoAl-LDH showed an obvious separation with the CoAl-LDH layer (pink) and Co-MOF layer (purple) shown in Figure S2b. Through zeta potential analysis, we further confirmed that both Co-MOF and CoAl-LDH show strong positive charges of +127.9 ± 0.6 mV and +74.9 ± 5.6 mV, respectively (Figure S3). As a result, we rationalize that the CoAl-LDH and Co-MOF would not form a homogeneous composite due to the repulsive electrostatic reaction. The SEM image of the mixture of Co-MOF and CoAl-LDH further proves that Co-MOFs and CoAl-LDH sheets are prone to remain separate rather than chemically-bonding together (Figure S4).

XRD was used to examine the crystal structure of CoAl-LDH, Co-MOF, and MOF/LDH/MOF. Figure 1d shows the XRD patterns of CoAl-LDH, Co-MOF and MOF/LDH/MOF. The diffraction peaks centered at 2*θ* = 11° and 23° indicate the characteristic (003) and (006) crystalline planes of CoAl-LDH, respectively [22]. The XRD pattern of Co-MOF shows characteristic diffraction peaks of (011), (002) and (012) planes at 2*θ* = 7°, 10°, 12°, respectively [23]. Accordingly, the MOF/LDH/MOF hybrid exhibits characteristic peaks for a combination of both CoAl-LDH sheets and Co-MOF crystals. We further analyzed the cross-sectional microstructure and its element analysis of the MOF/LDH/MOF by FEG-TEM (Figure 1e and Figure S5a,b). The element distribution of the MOF/LDH/MOF in Figure 1f–h shows that the elements Co, Al, and O are mostly concentrated in the middle of the sandwiched MOF/LDH/MOF hybrid, indicating that the CoAl-LDH sheets are embedded in the entire hybrid. In addition, Figure 1i–j shows that the elements C and N are distributed outside the CoAl-LDH sheets. This confirms the successful synthesis of the MOF/LDH/MOF hybrid structure via in-situ growth.

### 3.2. Formation and Characterizations of WNPC

**Figure 2:**
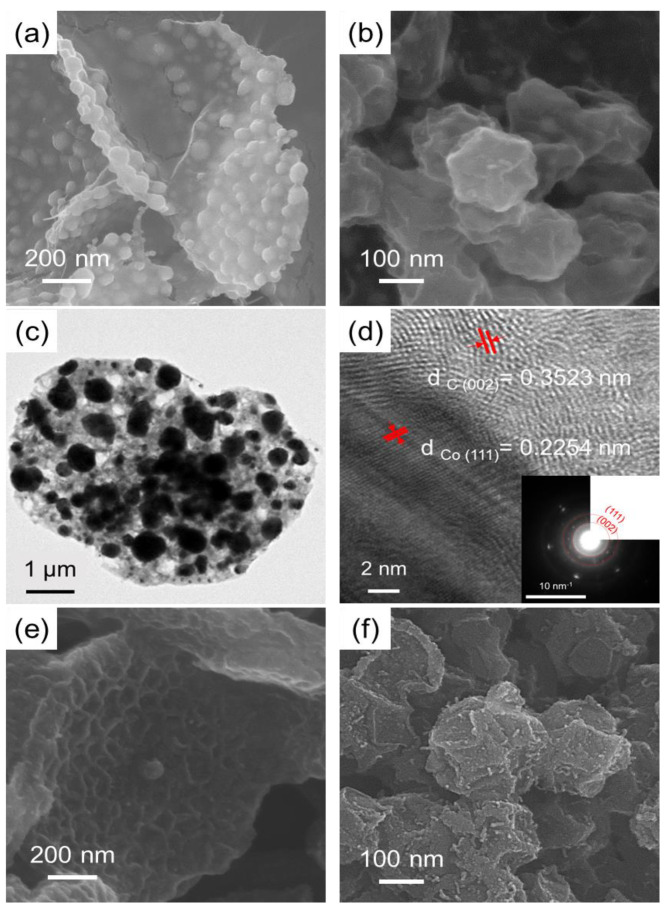
SEM images of (**a**) C_M/L/M_; (**b**) C_Co-MOF_; FEG-TEM image of (**c**) C_M/L/M_ and (**d**) the magnified FEG-TEM image with SAED in the inset; (**e**) WNPC and (**f**) nanoporous carbon (NPC).

Figure 2a shows the morphology of C_M/L/M_ (the carbonized sample of the MOF/LDH/MOF hybrid)_._ The C_M/L/M_ retained its structural integrity in the MOF/LDH/MOF hybrid under harsh pyrolysis at 800 °C. This can be attributed to the strong chemical bonding between Co-MOF crystals and CoAl-LDH sheets. Carbonized Co-MOF particles with irregular sizes were observed on the C_M/L/M_ carbon surface, consistent with the morphology of C_Co-MOF_ (Figure 2b). To further examine the crystalline nanostructure of C_M/L/M_, high-resolution FEG-TEM investigation was used (Figure 2c,d). The lattice fringes of 0.2254 nm and 0.3523 nm correspond to the (111) plane of metallic Co aggregates and the (002) plane of graphitic carbon layers, respectively, in the selected area electron diffraction pattern (SAED) [24,25]. Furthermore, the dark field of the TEM image and EDS elemental mapping in Figure S6a–g show that elements C, N, O, and Al were well distributed in C_M/L/M_ and aggregated Co metallic particles. It is worthy of note that the metallic Co aggregates are prone to being surrounded by graphitic carbons, a factor attributed to the aggregated Co, converted from Co-MOFs during pyrolysis, which provide a catalytic ability that induces crystalline carbon [19]. To obtain nanoporous carbons, we performed wet acid etching using phosphoric acid/hydrochloric acid solution to remove aggregated metals, and obtain WNPC afterwards (Figure 2e) [26,27]. In comparison with WNPC, we also prepared MOF-derived carbon (NPC) from the C_Co-MOF_ (the carbonized sample of Co-MOF) after acid etching (Figure 2f).

**Figure 3:**
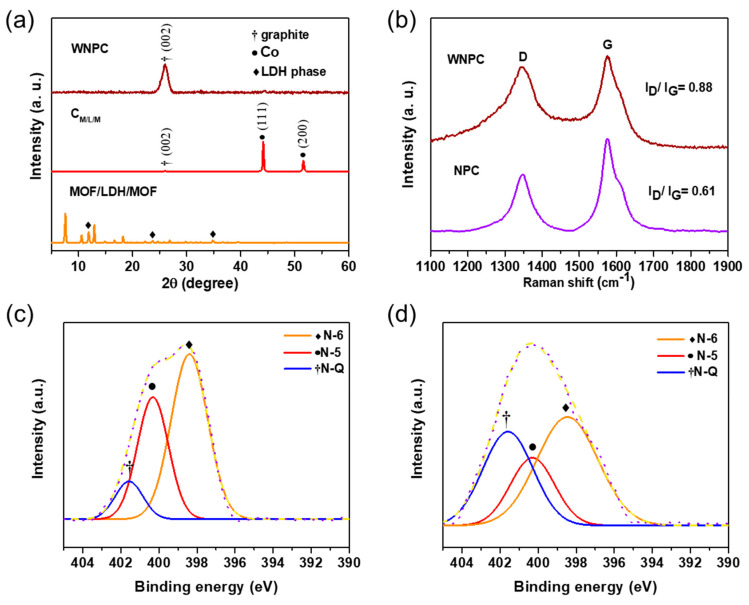
(**a**) XRD pattern of MOF/LDH/MOF, C_M/L/M_, and WNPC; (**b**) Raman spectrum of WNPC and NPC. High-resolution XPS spectra for N1s of (**c**) WNPC and (**d**) NPC.

XRD was again performed to analyze the crystal structure of C_M/L/M_ and WNPC. Figure 3a presents the XRD pattern of MOF/LDH/MOF, C_M/L/M_ and WNPC. The peak of C_M/L/M_ centered at 2*θ* = 26° indicated the graphite phase. Aside from this peak, two characteristic peaks are observed at 2*θ* = 44.1° and 2*θ* = 51.8°, corresponding to the diffraction peaks of metallic Co aggregates [28,29,30,31]. This is also consistent with the SAED results (Figure 2d). After the removal of metal aggregates by acid etching, the XRD pattern of WNPC shows only a graphitic crystalline peak centered at 2*θ* = 26°, proving that there are no metal aggregates left. Raman spectra were further used to examine the carbon structure of WNPC and NPC. In Figure 3b, both WNPC and NPC show two characteristic peaks of D band at ≈1347.1 cm^−1^ and G band at ≈1575.6 cm^−1^, indicating the features of disordered carbon (defective graphic structure) and graphite layers, respectively [32]. The intensity ratio (I_D_/I_G_) is commonly used to predict the degree of defects in carbon materials. The I_D_/I_G_ ratios of WNPC and NPC are 0.88 and 0.61, respectively, showing that WNPC exhibited a defective crystal nature due to the abundant heteroatom doping.

To verify this, we further examined the nitrogen chemical structure of WNPC and NPC using XPS (Figure 3c,d). The XPS survey spectra, shown in Figure S7 and Table 1, summarize the weight percentage of the elements C, N and O, where the carbonized sample of the mixture of Co-MOF and CoAl-LDH was also made for comparison. The high N content of 7.5 wt% in the WNPC is much higher than the 3.4 wt% in NPC and the 3.2 wt% of the carbonized sample of the mixture of Co-MOF and CoAl-LDH, a factor attributed to the fact that nitrogen atoms are likely to remain in the aromatic carbon ring instead of being lost because of the enhanced thermal stability by in-situ growth. Next, we divided the high-resolution N1s peak into several peaks including N-6 pyridine nitrogen (398.4 eV), N-5 pyrrole nitrogen (400.3 eV), and N-Q quaternary nitrogen (401.5 eV) to determine their respective fractions by calculating the integration areas of the respective peaks [33,34,35]. The fractions of N-6, N-5 and N-Q were 55.4%, 34.9%, and 9.7% for WNPC, which are believed to exhibit enhanced pseudocapacitance for electrochemical performance [36].

**Figure 4:**
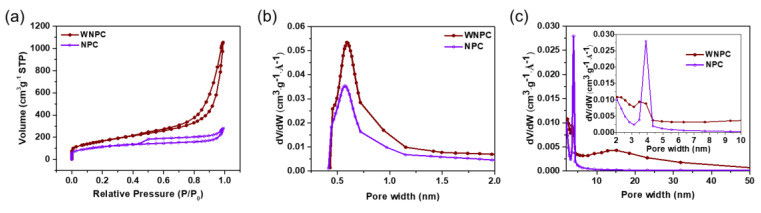
(**a**) N_2_ absorption–desorption isotherms, (**b**) the pore size distribution curve calculated by Brunauer, Emmett and Teller (BJH) method (slit-like pore) and (**c**) the pore size distribution curve calculated using the Horvath–Kawazoe (H–K) method (slit-like pore) of WNPC and NPC.

The specific surface areas and pore size distribution of WNPC and NPC were investigated by N_2_ adsorption–desorption isotherms (Brunauer–Emmett–Teller (BET) theory calculation). In Figure 4a, WNPC and NPC both presented H3 and H4 hysteresis loops, respectively [37]. According to the micropore distribution analysis (Figure 4b), the size distributions below 2 nm of NPC and WNPC are similar due to their intrinsic micropore nature. It is notable that WNPC exhibited a broader mesopore distribution of 2–50 nm (Figure 4c), which differed from the typical mesopore distribution of MOF [19], a factor attributed to the well-dispersed MOFs in the in-situ growth preventing the aggregation of Co-MOFs during carbonization. Therefore, WNPC also displayed a larger specific surface area (594 m^2^g^−1^) than NPC (394 m^2^g^−1^).

### 3.3. Electrochemical Performances of WNPC

**Figure 5:**
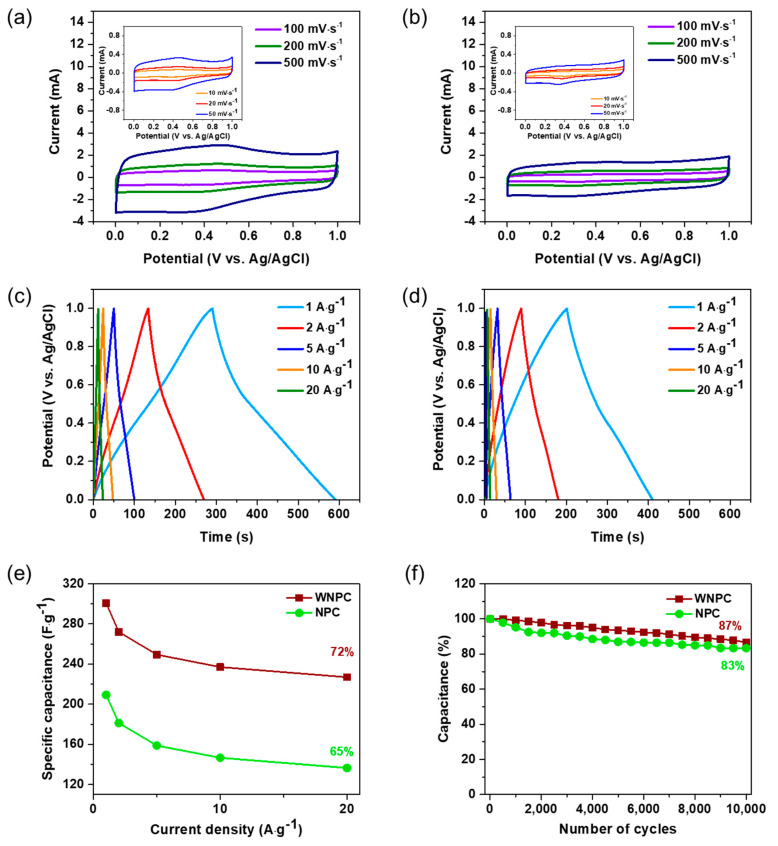
Cyclic voltammograms (CV) curves of (**a**) WNPC and (**b**) NPC with different scan rates; galvanostatic charge–discharge (GCD) curves of (**c**) WNPC and (**d**) NPC at different current densities; (**e**) Variation of specific capacitance against the current density of WNPC and NPC; (**f**) Cycling performance of WNPC and NPC electrode at a current density of 4 A·g^−1^.

The electrochemical performance of WNPC and NPC as supercapacitor electrodes was tested with a standard three-electrode system using 1 M H_2_SO_4_ solution. Figure 5a,b shows CV profiles of WNPC and NPC with scan rates ranging from 10 to 500 mV·s^−1^. For WNPC, broad humps appear at 0–0.4 V in the reductive sweep and at 0.4–0.8 in the oxidative sweep, representing pseudocapacitance by heteroatom-doping (N and O doping) [38]. Compared to the WNPC, the capacitive box of NPC behaves similarly to a typical EDLC with less of a heteroatom effect. The GCD profiles of WNPC and NPC in Figure 5c,d show that the specific capacitances of WNPC and NPC as supercapacitor electrodes are 300.7, 272.0, 249.4, 237.0, 226.9 F·g^−1^ and 209.2, 181.3, 158.8, 146.6, 136.4 F·g^−1^ at current densities of 1, 2, 5, 10, and 20 A·g^−1^, respectively. WNPC exhibits a higher capacitance retention of 72% compared to NPC of 65%, at the current density of 20 A·g^−1^ to 1 A·g^−1^ (Figure 5e). This result indicates that WNPC has faster electrode kinetics, a factor that might be consistent with the lesser aggregated structure, leading to better electrolyte migration. Table S1 summarizes the comparisons of the specific capacitance with different MOF-derived nanoporous carbons as supercapacitor electrodes from the previous studies. Our WNPC displays a higher specific capacitance at similar current densities. We performed GCD cycles at a current density of 4 A·g^−1^ to test the cycle stability of WNPC and NPC, as shown in Figure 5f. After 10,000 cycles, WNPC retained 87% of the initial value of the capacitor, which is higher than 83% of the NPC. This indicates that the WNPC shows excellent electrochemical cycle stability as a potential supercapacitor electrode.

**Figure 6:**
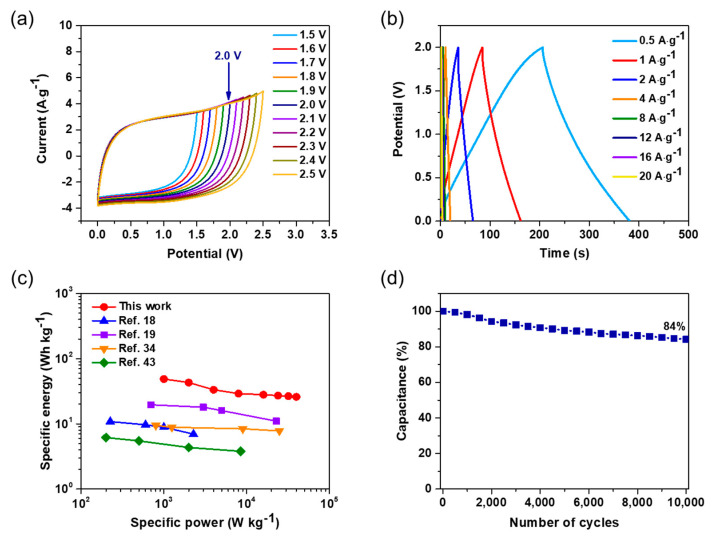
(**a**) CVs of the symmetric supercapacitor in two-electrode system with varying potential 1.5–2.5 V at a scan rate of 500 mV·s^−1^; (**b**) GCD curves with varying current densities from 0.5 to 20 A·g^−1^; (**c**) Ragone plot showing the comparison of our work with other MOF-derived nanoporous carbon based symmetric supercapacitors; (**d**) Cycle stability performance for the capacitance retention of WNPC symmetric supercapacitor.

For further practical demonstration of WNPC as an electrode material for supercapacitors, we constructed a two-electrode symmetrical supercapacitor cell in a 1 M TEABF_4_ organic electrolyte. The schematic illustration of the coin cell device is shown in Figure S8. The WNPC-based supercapacitor presents a regular rectangular CV shape measured by applying a potential varying from 1.5 to 2.5 V at a san rate of 500 mV·s^−1^ (Figure 6a), which illustrates that the voltage window of 1.5–2.5 V is feasible for a WNPC-based supercapacitor with a TEABF_4_ organic electrolyte. In order to avoid unwanted electrolyte decomposition, we adopted the operational potential of 2 V as demonstrated. GCD measurements performed from 0.5 to 4 A·g^−1^ for the WNPC-based supercapacitor are displayed in Figure 6b. The capacitance retention of the WNPC-based coin cell supercapacitor at different current densities is shown in Figure S9. The Ragone plot, correlating the specific energy with the specific power, shows a comparison with our WNPC-based supercapacitor with other MOF-derived nanoporous carbons as full cell symmetric supercapacitors (Figure 6c). The WNPC-based supercapacitor can reach the specific energy of 27 W·h·kg^−1^ with a specific power of 500 W·kg^−1^ at 0.5 A·g^−1^, and keeps a high specific energy of 19.7 W·h·kg^−1^ even with a large specific power of 4000 W·kg^−1^ at 4 A·g^−1^, which is much higher than those reported for MOF-derived nanoporous carbon based supercapacitors. Finally, cycle stability performance of this WNPC-based device was measured and it displays a high capacitance retention of ~85% at a current density of 4 A·g^−1^ for 10,000 cycles (Figure 6d).

## 4. Discussion

Pore engineering is an effective approach to enhance the electrochemical performance of carbon based materials. In general, the capacitance of supercapacitors is a function of the specific surface areas [39]. However, for a typical activated carbon (AC) material, with specific surface area in the range of 1000 to 2000 m^2^g^−1^ or even higher and pore size distribution of 2–5 nm, the gravimetric capacitance has been measured at about 100 to 120 F·g^−1^ in organic electrolytes, which is not as good as expected due to the poor efficiency of electrolyte migration from mesopores to micropores [40]. Therefore, it is critical to develop a unique micro/meso/macro interconnected porous hierarchical carbon structure, where macropores could serve as reservoirs for electrolyte ions, mesopores could offer fast ionic transportation, and micropores could provide a high specific surface area for the adsorption/desorption of electrolyte ions [41].

MOFs with an extreme high specific surface area have been suggested as potential materials for supercapacitor electrodes but have been limited for nanoporous hydrolyzed carbons [18], as they lead to a much lower electrical conductivity. Although carbonized MOFs can increase the electrical conductivity by graphitization at a higher temperature pyrolysis, they are usually accompanied with severe aggregation and reduced N-doped yields due to low C–N binding energy [17]. We utilized dual-atom cobalt aluminum layered double hydroxide (CoAl-LDH) as a synthetic template for the in-situ growth of Co-MOF onto CoAl-LDH to obtain waffle-like nanoporous carbons (WNPC). The element distribution of MOF/LDH/MOF shows that the elements Co, Al, and O mostly concentrated in the middle of the sandwiched MOF/LDH/MOF, confirming the successful synthesis of the MOF/LDH/MOF hybrid structure via in-situ growth. The formation of the waffle-like morphology can be explained as follows: (1) In the beginning, as the MOF/LDH/MOF was pyrolyzed, Co-MOF collapsed and started to melt and connect together. (2) Meanwhile, Co atoms began to aggregate, owing to their intrinsic affinity, thus leading to a strong graphitization around the Co aggregates. (3) The nitrogen-containing carbon compounds from the collapsed Co-MOF bridged together around the Co aggregates and gradually merged into a network. Finally, the WNPC was fabricated by removing the Co aggregates and aluminum oxides using acid etching.

In our work, by controlling the intimate chemically-bonded nature between MOFs and LDH sheets via the in-situ reaction, heteratoms were strongly anchored in the carbon network rather than leaking out during harsh pyrolysis, thus leading to a higher nitrogen and oxygen retention. The N- and O-doped heteroatoms in WNPC both contributed in pseudocapacitance, a factor found in the broad humps in the CV profile [38,42]. Note that the oxygen content originated from the rich oxygen-containing groups in LDH. Furthermore, since well-ordered Co atoms on CoAl-LDH serve as nucleation and growth sites, the narrow slit-like pore nature of MOFs is likely to rearrange in a well ordered fashion, instead of forming agglomerated carbonized particles, and thus creates abundant mesopores interconnected with micropores originating from MOFs, which promotes a sieving effect that allows efficient ion migration from mesopores into micropores. As a result, the waffle-like carbon network obtained by the in-situ nucleation strategy and direct growth implements a promoted micro/mesoporous hierarchical structure for the effective electrolyte migration [43,44].

The 2D carbon-based framework has been successfully achieved by heteroatom and pore engineering, and can be extended to the preparation of other LDH/MOF derived functional materials toward energy storage. The WNPC-based supercapacitor exemplifies the specific energy of 27 W·h·kg^−1^ with a specific power of 500 W·kg^−1^ at 0.5 A·g^−1^, and maintains a high specific energy of 19.7 W·h·kg^−1^ even with a large specific power of 4000 W·kg^−1^ at 4 A·g^−1^. The WNPC-based device displayed better cycle stability performance with a high capacitance retention of ~85% at a current density of 4 A·g^−1^ for 10,000 cycles.

## 5. Conclusions

We successfully synthesized a sandwich MOF/LDH/MOF hybrid by an in-situ growth method. After pyrolysis and acid etching, a unique waffle-like carbon structure with high heteroatom-doped (WNPC) was formed. Furthermore, WNPC possesses a rich and broader distribution of mesoporous structure, which helps the migration of electrolyte diffusion from mesopores to micropores due to the sieving effect. The resulting high-nitrogen (7.5 wt%) and high-oxygen content (9.1 wt%) of WNPC with meso/microporous hierarchical characteristics contributed to a good specific capacitance of 300.7 F·g^−1^ at 1 A·g^−1^ in 1 M H_2_SO_4_. The WNPC-based symmetric coin cell supercapacitor reaches a superior specific energy of 27 W·h·kg^−1^ at a specific power of 500 W·kg^−1^ with the voltage window of 2 V, and shows good cycling durability (85% capacitance retention after 10,000 cycles).

## Figures and Tables

**Table 1:** Elemental analysis results for NPC, WNPC and the carbonized sample of the mixture of Co-MOF and CoAl-LDH.

Sample	C (wt%)	N (wt%)	O (wt%)	Pyridinic N (%)	Pyrrolic N (%)	Quaternary Nitrogen (%)
WNPC	83.4	7.5	9.1	55.4	34.9	9.7
NPC	94.1	3.4	2.5	46.4	20.9	32.7
Mixture of Co-MOF and CoAl-LDH	58.7	3.2	38.1	30.5	22.1	47.3

## Supplementary Materials

The following are available online at https://www.mdpi.com/2079-4991/10/12/2388/s1, Figure S1: SEM low magnification image of the MOF/LDH/MOF hybrid. Figure S2. Optical images of (a) MOF/LDH/MOF hybrid powders after centrifugation and (b) The mixture of CoAl-LDH and Co-MOF after centrifugation. Figure S3. Zeta potential of CoAl-LDH and Co-MOF in water at pH=7. Figure S4. SEM image of the mixture of CoAl-LDH and Co-MOF powders. Figure S5. The cross-sectional image of (a) MOF/LDH/MOF hybrid observed and sampled by FIB and (b) the FEG-TEM low-magnified image of MOF/LDH/MOF hybrid. Figure S6. Dark-field FEG-TEM image of (a) CM/L/M and (b–f) the corresponding element mapping images of elements C, N, O, Co and Al. (g) The overlapped element mapping. Figure S7. The XPS surveys of WNPC, NPC and the mixture of CoAl-LDH and Co-MOF derived carbon. Figure S8. Schematic illustration of WNPC based coin cell. Figure S9. Specific capacitance variation of the WNPC coin cell at different current densities. Table S1: Capacitances in aqueous electrolytes of various porous carbons reported in the representative literatures.
