# Supplementary material for: Waffle-Like Carbons Combined with Enriched Mesopores and Highly Heteroatom-Doped Derived from Sandwiched MOF/LDH/MOF for High-Rate Supercapacitor
Source: Nanomaterials (Basel). 2020 Nov 30;10(12):2388. doi: 10.3390/nano10122388 (PMC7760639; doi:10.3390/nano10122388)
Supplement: Supplementary file 1 [file nanomaterials-10-02388-s001.pdf]

nanomaterials

Supplementary Material

Waffle-Like Carbons Combined with Enriched Mesopores and Highly Heteroatom-doped Derived from Sandwiched MOF/LDH/MOF for High-rate Supercapacitor

Szu-Chen Wu, Po-Hsueh Chang, Syun-Hong Chou, Chih-Yang Huang, Ta-Chung Liu and Cheng-Hsiung Peng

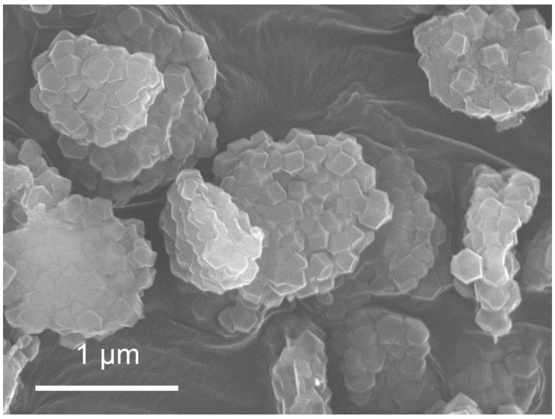

Figure S1. SEM low magnification image of the MOF/LDH/MOF hybrid.

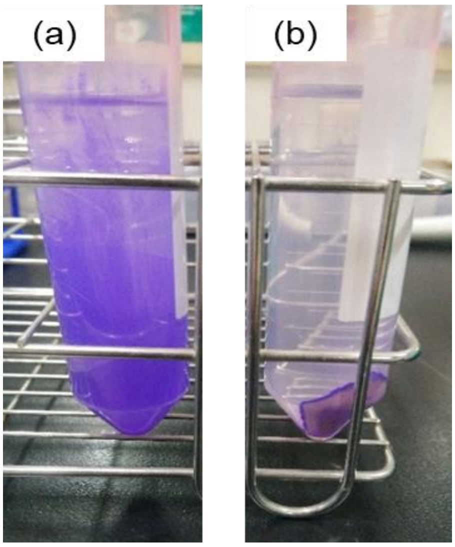

Figure S2. Optical images of (**a**) MOF/LDH/MOF hybrid powders after centrifugation and (**b**) The mixture of CoAl-LDH and Co-MOF after centrifugation.

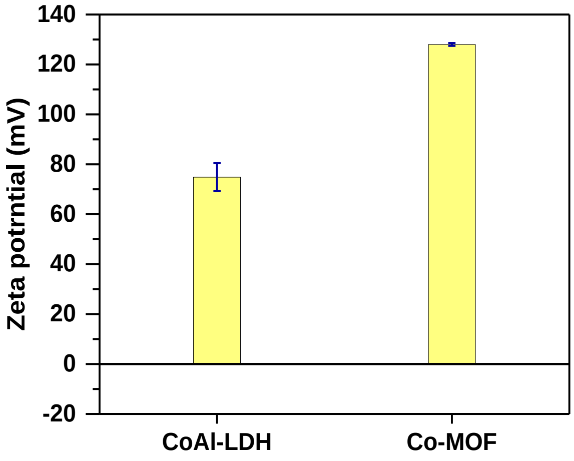

Figure S3. Zeta potential of CoAl-LDH and Co-MOF in water at pH=7.

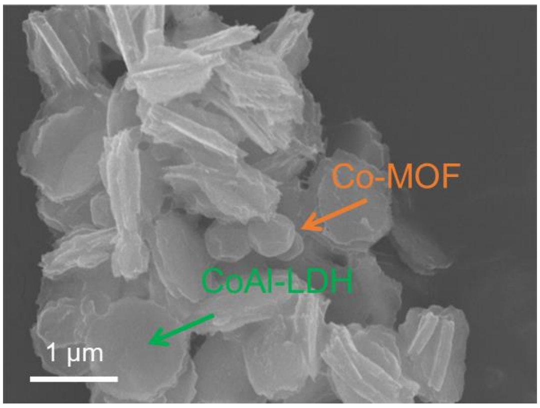

Figure S4. SEM image of the mixture of CoAl-LDH and Co-MOF powders.

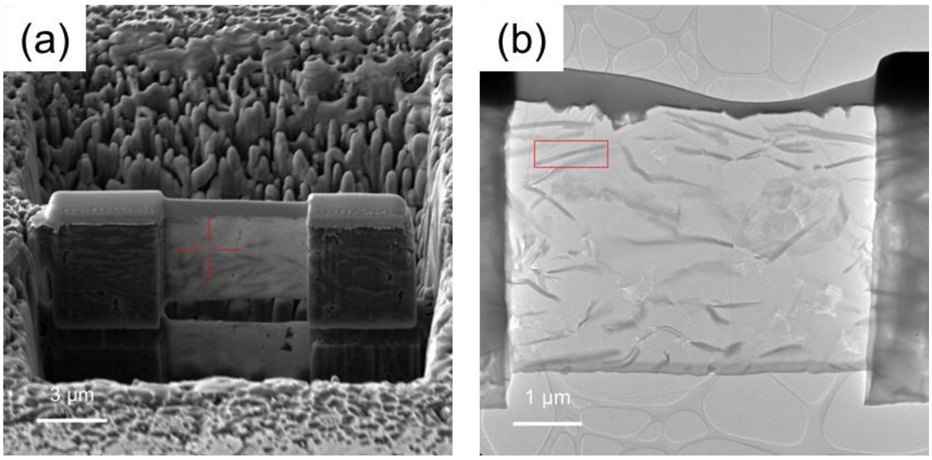

Figure S5. The cross-sectional image of (**a**) MOF/LDH/MOF hybrid observed and sampled by FIB and (**b**) the FEG-TEM low-magnified image of MOF/LDH/MOF hybrid.

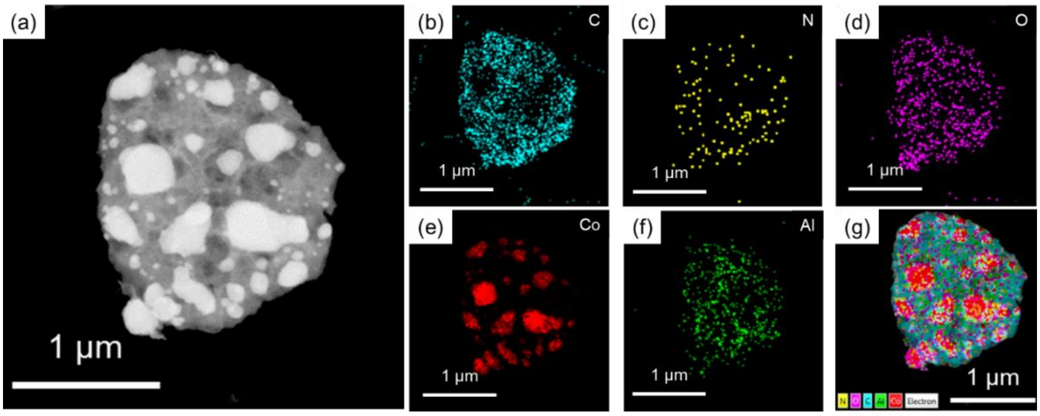

Figure S6. Dark-field FEG-TEM image of (**a**) $C_{M/L/M}$ and (**b**–**f**) the corresponding element mapping images of elements C, N, O, Co and Al. (**g**) The overlapped element mapping.

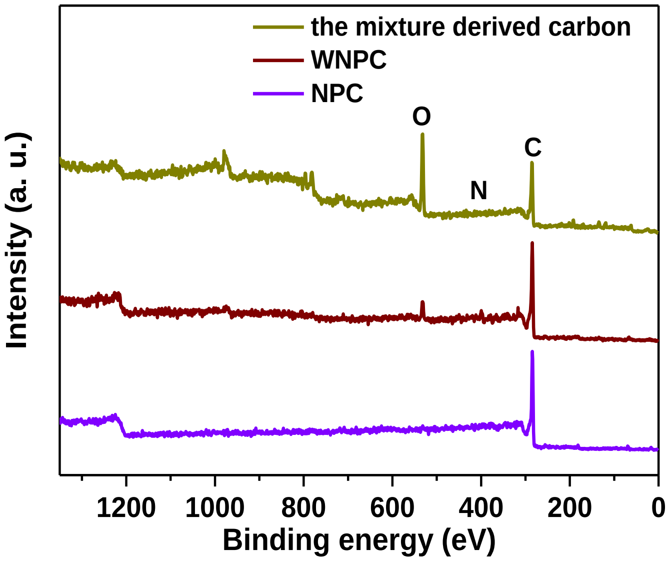

Figure S7. The XPS surveys of WNPC, NPC and the mixture of CoAl-LDH and Co-MOF derived carbon.

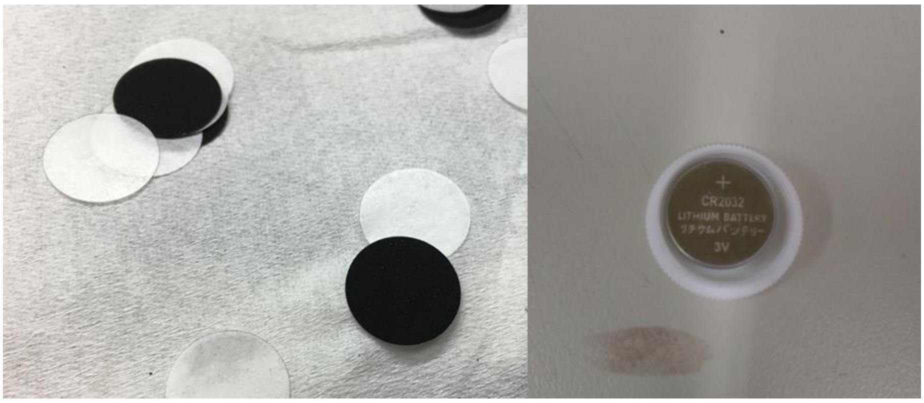

Figure S8. Schematic illustration of WNPC based coin cell.

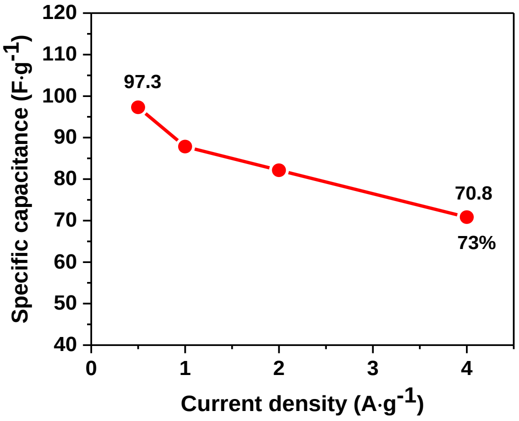

Figure S9. Specific capacitance variation of the WNPC coin cell at different current densities.

Table S1. Capacitances in aqueous electrolytes of various porous carbons reported in the representative literatures.

Materials	N content	Electrolyte	Capacitance ($F{\cdot}g^{-1}$)	Ref.
		MOF derived Nanoporouscarbon		
WNPC	8 wt%	1 M H_2SO_4	300.7 at 1 $A{\cdot}g^{-1}$; 240.1 at 10 $mV{\cdot}s^{-1}$	This work
NC@GC(0.05) [a]	10.6 at%	1 M H_2SO_4	200 at 4 $A{\cdot}g^{-1}$	[1]
C1000 [b]	-	1 M H_2SO_4	200 at 0.25 $A{\cdot}g^{-1}$	[2]
CZIF69a [c]	1.2 wt%	0.5 M H_2SO_4	162 at 10 $mV{\cdot}s^{-1}$	[3]
Z-900 [d]	-	0.5 M H_2SO_4	214 at 5 $mV{\cdot}s^{-1}$	[4]
AS-ZC-800 [e]	-	1.0 M H_2SO_4	211 at 10 $mV{\cdot}s^{-1}$	[5]

[a] core-shell ZIF-8@ZIF-67 (zeolite imidazole framework, ZIF; ZIF-8 and ZIF-67 is Zn based and Co based and 2-methylimidazole ligand) (Co^{2+}/Zn^{2+} ratio 0.05) derived NC@GC (nitrogen-doped carbon@graphitic carbon) (0.5); [b] C1000: carbonized FA (furfuryl alcohol)/ ZIF-8 composite at 1000 °C; [c] CZIF69a: carbonized ZIF-69 where KOH as an active agent; [d] Z-900: carbonized ZIF-8 at 900 °C; [e] AS-ZC-800: carbonized ZIF-8 where KOH as an active agent.

References

1. Tang, J.; Salunkhe, R.R.; Liu, J.; Torad, N.L.; Imura, M.; Furukawa, S.; Yamauchi, Y. Thermal Conversion of Core–Shell Metal–Organic Frameworks: A New Method for Selectively Functionalized Nanoporous Hybrid Carbon. *J. Am. Chem. Soc.* **2015**, *137*, 1572–1580.
2. Jiang, H.L.; Liu, B.; Lan, Y.Q.; Kuratani, K.; Akita, T.; Shioyama, H.; Zong, F.Q.; Xu, Q. From Metal-Organic Framework to Nanoporous Carbon: Toward a Very High Surface Area and Hydrogen Uptake. *J. Am. Chem. Soc.* **2011**, *133*, 11854–11857.
3. Wang, Q.; Xia, W.; Guo, W.; An, L.; Xia, D.; Zou, R. Functional Zeolitic-Imidazolate-Framework-Templated Porous Carbon Materials for CO_2 Capture and Enhanced Capacitors. *Chem. Asian J.* **2013**, *8*, 1879–1885.
4. Chaikittisilp, W.; Hu, M.; Wang, H.; Huang, H.-S.; Fujita, T.; Wu, K.C.W.; Chen, L.-C.; Yamauchi, Y.; Ariga, K. Nanoporous carbons through direct carbonization of a zeolitic imidazolate framework for supercapacitor electrodes. *Chem. Commun.* **2012**, *48* (58), 7259–7261.
5. Amali, A.J.; Sun, J.-K.; Xu, Q. From assembled metal–organic framework nanoparticles to hierarchically porous carbon for electrochemical energy storage. *Chem. Commun.* **2014**, *50*, 1519–1522.

Publisher's Note: MDPI stays neutral with regard to jurisdictional claims in published maps and institutional affiliations.

© 2020 by the authors. Submitted for possible open access publication under the terms and conditions of the Creative Commons Attribution (CC BY) license (http://creativecommons.org/licenses/by/4.0/).
